# A Treaties on the Diseases of the Eye

**Published:** 1854-07

**Authors:** 


ART VI.—*A Treatise on the Diseases of the Eye.* By W. LAWRENCE, F. R. S., Surgeon extraordinary to the Queen; Surgeon to St. Bartholomews Hospital, and Lecturer on Surgery to that Hospital; Surgeon to Bethlem and Bridewell Hospitals; and late Surgeon to the Ophthalmic Infirmary. A new edition, edited, with numerous additions and 243 illustrations, by ISAAC HAYS, M. D., Surgeon to Wills Hospital; Fellow of the Philadelphia College of Physicians; Member of the American Medical Association; of the American Philosophical Society; of the Academy of Natural Sciences of Philadelphia, &c., &c. Philadelphia: Blanchard & Lea, 1854; p. 948, 8vo.

The reader will recognize in the above the title of a work which has long stood at the head of the Surgical literature relating to the Eye. The author is one of the most eminent of living surgeons, and his authority on all subjects to which his attention has been given, the highest. Dr. Hays has undertaken to prepare a new edition of the work, the author having declined the task at his advanced age. In the performance of his task, the editor has retained the whole of the original, at the same time that he has incorporated such improvements and discoveries as have been made within a few years in ophthalmic pathology and practice. Among the additions which have been made, he mentions, a full account of the recent microscopical investigations into the structure and pathology of the eye; the descriptions of various affections not treated of in the original; an account of the catoptric examination of the eye, and of its employment as a means of diagnosis; a description of recently invented instruments for illuminating the retina, and of some new methods of examining the interior structure of the eye, and the illustrations and full index.

The healing art is not improving more rapidly in any direction than in its mechanical means of diagnosis. Since the introduction of the new era by the application of physical processes to affections of the chest we have been steadily advancing in the same line of improvement. Similar methods have been adopted for detecting the morbid states of the uterus, and in the diagnosis of diseases of the eyes we are deriving important aid from mechanical appliances. The first step towards effecting a cure, undoubtedly, is to ascertain with accuracy the nature of the morbid condition, and this we are doing by the application of the principles of physical science where they are available in diagnosis. The eye is precisely the organ to which, from being external and transparent, we should expect these principles would be most successfully applied.

The eye and its diseases form a most interesting specialty in our profession, and pretenders are reaping a rich harvest from it. The subject is one which demands much study, and in its prosecution we do not know so complete and trustworthy a manual as this elaborate treatise by Mr. Lawrence.

